# Supplementary material for: An album is a story: Feature arcs in sequences of tracks
Source: PLoS One. 2025 Jul 3;20(7):e0316963. doi: 10.1371/journal.pone.0316963 (PMC12225790; doi:10.1371/journal.pone.0316963)
Supplement: S1 — Details of the musical tracks used in the study, including album names, artist names, dataset sources, and track correspondence. Includes participant musical experience data summarized in tables (PDF) [file pone.0316963.s001.pdf]

S1 Appendix. Track information.

Songs from Set 1

- **Album name**: Manhã
- **Artist names**: Lupa Santiago & Paulo Braga
- **Track correspondence**: Track 1 - Dom do samba; Track 2 - 2 de Janeiro; Track 3 - Oolaia; Track 4 - Manhã; Track 5 - Prolífero & Perdulário
- **Available at**: https://open.spotify.com/album/1HhW59gaKkfq0olN8Hd3WP

Songs from Set 2

- **Dataset**: Dataset on Induced Musical Emotion from Game with a Purpose Emotify
- **Track ID (as in published dataset)**: Track 1 - 18.mp3; Track 2 - 39.mp3; Track 3 - 55.mp3; Track 4 - 78.mp3; Track 5 - 82.mp3
- **Available at**: http://www2.projects.science.uu.nl/memotion/emotifydata/

Songs from Set 3

- **Dataset**: Dataset on Induced Musical Emotion from Game with a Purpose Emotify
- **Track ID (as in published dataset)**: Track 1 - 30.mp3; Track 2 - 40.mp3; Track 3 - 45.mp3; Track 4 - 54.mp3; Track 5 - 99.mp3
- **Available at**: http://www2.projects.science.uu.nl/memotion/emotifydata/
