# Supplementary material for: An album is a story: Feature arcs in sequences of tracks
Source: PLoS One. 2025 Jul 3;20(7):e0316963. doi: 10.1371/journal.pone.0316963 (PMC12225790; doi:10.1371/journal.pone.0316963)
Supplement: S2 — Mean and standard deviation (in parentheses) of albums recorded, number of concerts performed, and years of experience as professional musicians across the three track sets. (PDF) [file pone.0316963.s002.pdf]

S2 Participant's musical experience

Table 1: **Participants' musical experience.** Mean and standard deviation (in parentheses) of albums recorded, number of concerts, and years as professional musicians across the three sets.

Set	Albums Recorded	Number of Concerts	Years as Professional
1	3.71 (4.07)	95.5 (200.4)	13.36 (8.98)
2	4.53 (9.86)	10.42 (12.15)	12.97 (10.26)
3	2.16 (1.77)	183.8 (369.24)	13.52 (13.55)
