# Supplementary material for: An album is a story: Feature arcs in sequences of tracks
Source: PLoS One. 2025 Jul 3;20(7):e0316963. doi: 10.1371/journal.pone.0316963 (PMC12225790; doi:10.1371/journal.pone.0316963)
Supplement: S3 — Chi-square and Dip-test statistics and their corresponding p-values for each survey proposition. (PDF) [file pone.0316963.s003.pdf]

S3 Detailed Survey Statistics

Table 1: **Survey statistical tests.** Chi-square and Dip-test statistics and their corresponding p-values for each proposition.

	Chi-square		Dip-test	
Proposition	Statistic	p	Statistic	p
A	152.53	0.00***	0.09	0.00***
B	52.65	0.00***	0.08	0.00***
C	16.14	0.00***	0.09	0.00***
D	1.75	0.42	0.11	0.00***
E	20.42	0.00***	0.10	0.00***
F	10.60	0.00***	0.07	0.00***
G	6.71	0.03*	0.08	0.00***
H	18.47	0.00***	0.09	0.00***
I	11.02	0.00***	0.07	0.00***
