# Supplementary material for: An album is a story: Feature arcs in sequences of tracks
Source: PLoS One. 2025 Jul 3;20(7):e0316963. doi: 10.1371/journal.pone.0316963 (PMC12225790; doi:10.1371/journal.pone.0316963)
Supplement: Attachment — Submitted filename: rebuttal_letter.pdf [file pone.0316963.s004.pdf]

Rebuttal letter

Dear editor,

We have now completed the revision of our paper, and we believe to have satisfied all the required changes and additions to our submission. Find below our responses (highlighted in yellow) to each point raised.

Best regards,
P.N.

1. Please ensure that your manuscript meets PLOS ONE's style requirements, including those for file naming. The PLOS ONE style templates can be found at
https://journals.plos.org/plosone/s/file?id=wjVg/PLOSOne_formatting_sample_main_body.pdf and
https://journals.plos.org/plosone/s/file?id=ba62/PLOSOne_formatting_sample_title_authors_affiliations.pdf

- We made the necessary adjustments in the formatting of our document. We have used the LaTex template, and now corrected the figure naming and author affiliation information;

2. Please note that PLOS ONE has specific guidelines on code sharing for submissions in which author-generated code underpins the findings in the manuscript. In these cases, we expect all author-generated code to be made available without restrictions upon publication of the work. Please review our guidelines at https://journals.plos.org/plosone/s/materials-and-software-sharing#loc-sharing-code and ensure that your code is shared in a way that follows best practice and facilitates reproducibility and reuse.

- We made an additional commitment to share our code, and added it to the data and made it available in Open Science Framework (OSF). The DOI of this repository (http://doi.org/10.17605/OSF.IO/69N3X) is added to the manuscript. However, as is the case with the data, code components will only be made available upon the publication of our research.

3. Thank you for stating the following financial disclosure: "This research was funded by the Research Council of Finland, under the Center of Excellence in Music, Mind, Body and Brain (Project number: 346210)." Please state what role the funders took in the study. If the funders had no role, please state: "The funders had no role in study design, data collection and analysis, decision to publish, or preparation of the manuscript." If this statement is not correct you must amend it as needed. Please include this amended Role of Funder statement in your cover letter; we will change the online submission form on your behalf
.

- It is now stated that our funders had no role in the design, data collection, analysis, preparation and publication of our manuscript. This statement is added to the cover letter, as instructed;

4. Please note that your Data Availability Statement is currently missing the repository name and/or the DOI/accession number of each dataset OR a direct link to access each database. If your manuscript is accepted for publication, you will be asked to provide these details on a very short timeline. We therefore suggest that you provide this information now, though we will not hold up the peer review process if you are unable.

- We have amended the data availability statement, which now shows the DOI (http://doi.org/10.17605/OSF.IO/69N3X) of the OSF project containing all the data collected and analysed in our study, as well as the relevant code. Although the DOI is now available, the data component is still private, and will be made available immediately upon publication of our research;

5. When completing the data availability statement of the submission form, you indicated that you will make your data available on acceptance. We strongly recommend all authors decide on a data sharing plan before acceptance, as the process can be lengthy and hold up publication timelines. Please note that, though access restrictions are acceptable now, your entire data will need to be made freely accessible if your manuscript is accepted for publication. This policy applies to all data except where public deposition would breach compliance with the protocol approved by your research ethics board. If you are unable to adhere to our open data policy, please kindly revise your statement to explain your reasoning and we will seek the editor's input on an exemption. Please be assured that, once you have provided your new statement, the assessment of your exemption will not hold up the peer review process.

- We are able to adhere to the journal's open data policy, and we have stated that all the data will be available upon acceptance of our article. Now we have added the DOI where all our data will be shared without restriction. Is this what is meant by "data sharing plan"?

6. While revising your submission, please upload your figure files to the Preflight Analysis and Conversion Engine (PACE) digital diagnostic tool, https://pacev2.apexcovantage.com/

- Our images were submitted to PACE, and the updated submission contains the corrected versions of all images.

Reviewer's comments

7. One of the PLOS's core values is transparency. As an academic editor, it is my responsibility to ensure that all authors of the articles published in PLOS ONE have made the data underlying their findings fully available. Please take a look at our criteria for publication (https://journals.plos.org/plosone/s/criteria-for-publication). The current submission does not include where the data can be retrieved. The authors' input is simply a copy-and-paste of the sample text: 'The data underlying the results presented in the study are available from XXX'. Please see our resource (https://journals.plos.org/plosone/s/data-availability) to understand what constitutes the 'minimal data set' and ideas about sharing data.

- We have now changed our data availability statement, and we provide the specific DOI (http://doi.org/10.17605/OSF.IO/69N3X) where all the data and code will be available upon publication. For the time being, we provide the minimal data set as supporting information to our submission.
