# Supplementary material for: An album is a story: Feature arcs in sequences of tracks
Source: PLoS One. 2025 Jul 3;20(7):e0316963. doi: 10.1371/journal.pone.0316963 (PMC12225790; doi:10.1371/journal.pone.0316963)
Supplement: Attachment — Submitted filename: response_reviewers.pdf [file pone.0316963.s005.pdf]

Editor's comments

ANOVA only tests for the inequality of group means and does not support the findings of U or inverted-U patterns. Given that this is one of the main conclusions, it needs to be explicitly tested. To test the U or inverted U patterns, a quadratic fit can be compared with a linear fit using an F-test (e.g., general linear model).

> **Response:** Good point. We have now compared the residuals of a quadratic versus a linear fit using an F-test. Results indicate that the quadratic term significantly improves the fit of the model. *Results. Feature analysis* - paragraph 3.

It needs to be clarified that "valence" and "arousal" are only estimates (or predictions) from the MIR toolbox and not human-rated measures. The cited studies are not the validation of the MIR toolbox estimates (which should have been presented in this context), but the investigation of the psychological constructs ("valence" and "arousal") that were self-reported by human participants. However, readers may not know how good (or poor) the MIR toolbox estimates are in predicting human ratings. It must be clarified that the "valence" and "arousal" are not human ratings but models' predictions. Also, if possible, provide validation studies that support the validity of the MIR toolbox in terms of those features.

> **Response:** This has now been specified. *Materials and methods. Feature analysis* - paragraph 3.

The averaged l2-norm of the first-order difference of the feature vectors (or scalars) was defined as an "overall dissimilarity" measure and tested against the control condition (i.e., H0: random changes) [Figure 6]. But, it seems that the changes between particular positions were reported in the text (e.g., "P1/P2 = 3.15" [line 233]), not the "overall dissimilarity" measure, which should have been averaged across all transitions. This needs to be consistently reported.

> **Response:** In fact, our equation represented the average dissimilarity across all pairs of tracks in an album, while our results were showing feature dissimilarities for specific pairs of tracks. For consistency, we now changed equation 2 to represent the overall feature dissimilarity between a pair of tracks, rather than the overall album dissimilarity. *Materials and methods. Feature analysis* . Equation 2. We believe this to be more informative, as there could have been an effect for a particular pair of tracks (e.g., the last pair being more different than the other pairs). In any case, our results were null for dissimilarity values, regardless if the scores were aggregated or separate for each pair. We also noticed that the term "tempo difference" was contrasting with the other features, where we used "dissimilarity". This is now consistent throughout the paper.

When defining a distance metric based on Pearson correlation, 1-r is more conventional for its range will be positive [0, 2].

> **Response:** Changed. *Materials and methods. Feature analysis.* Paragraph 8

[line 193] It is unclear why the authors want to test "whether or not participants leaned toward one or more segments of the scale" [line 203]. As shown in [line 97], the survey was carried out to find what are the factors that the musicians would say. For example, "Do they think a good sequence is important or not". Now the authors are answering a different question: "Do they think sequence is uniformly important and unimportant?" But why do you want to know this instead of the first question? Is this a sign of p-hacking?

Response:

In order to answer the question *"Do musicians consider a good sequence important?"*, we believe it is essential to investigate the distribution of responses throughout different segments of the scale. Statistical methods must be appropriate for the nature of the data, and this choice cannot always be determined a priori.

In our case, the distribution of survey responses indicated potential polarization, which warranted an approach that does not rely on measures of central tendency. Previously, we relied only upon visual inspection, but now we support our decision with Hartigan's dip test, which shows that responses to our survey were indeed not uni-modal. Materials and Methods. Survey analysis. Paragraph 1; Supplementary Materials.

Intuitively, consider a scenario in which half of the musicians completely agree with a given statement, while the other half completely disagree. Statistical tests based on central tendency measures would suggest that responses are neutral while, in reality, they are highly polarized. This is explained in Materials and Methods. *Survey Analysis*, paragraph 2.

Regarding p-hacking, an alternative approach involving central tendency measures to assess the significance of survey responses would likely produce the same number of significant results as our Chi-squared analysis (as suggested by the distributions shown in *Figure 7, items A, B, E, F, and I*). However, we did not conduct these tests, as our approach was guided by theoretical considerations rather than by an attempt to find small p-values.

We also emphasize that this survey was included in our study as a way to provide an additional angle of exploration to song sequencing. Conclusions and comparisons that we make about survey results are not presented with an overly firm stance (e.g., *Results. Survey* - paragraph 5; *Discussion. Comparison between survey responses and track sequences* - paragraphs 1 and 7), and they do not interfere with the main findings of the paper. We have also added further clarification to our reasoning behind binning (*Materials and methods. Survey analysis*. paragraphs 1-3), and completely restructured the section *Materials and methods. Survey analysis*, to make everything clearer.

Generally binning needs to be better justified. The non-Gaussianity of the ordinal measure such as the Likert scale can be still dealt with by other non-parametric alternative approaches such as the rank-sum test.

Response: In fact, the normality of residuals was not our primary concern and, as you suggest, non-parametric methods could be used to test whether responses to a particular item differ significantly from 50, for instance. However, any approach based on measures of central tendency would fail to account for the multimodal nature of responses, as illustrated in the previously described scenario. We have now sought to clarify this reasoning in *Survey Analysis*, paragraphs 1 and 2.

Even if the binning can be justified, why unequal segments as [45%, 10%, 45%] instead of not [33%, 33%, 33%]?

Response: Our original intention was to convert responses to *agree* (>50), *disagree* (<50) and *neutral* (50). However, as the computer interface of the scale did not show the exact number that participants were choosing (Figure 2), it would be easy for someone to respond e.g. 51, when they actually meant 50, or

neutral. The middle category was chosen as 10% to account for these likely imprecisions. However, we realize that this is an arbitrary threshold, and we now follow your suggestion of equal-width binning, which is more intuitive and easier to justify. The new binning approach caused propositions G and H to become statistically significant, and proposition D became non-significant. Overall, we think this change of results has little implication to our study, as we do not pursue any deep line of inquiry regarding survey responses, and their possible comparison with sequencing regularities. *Materials and methods. Survey analysis* - paragraph 3.

Reviewer 1

I find the topic of the manuscript quite interesting, and while it was easy for me to understand why this topic is relevant from a theoretical standpoint (the lack of systematic investigations into the matter; p. 2, lines 32-33), I struggled to see the practical relevance of it. Would understanding specific sequencing principles help an album's total sales or streams? A similar practical point is mentioned in the Discussion with regards to story arcs in literary works (p. 9, lines 328-329), but not in the Introduction. I feel that the manuscript would benefit from a short elaboration of why understanding album sequencing principles would be practically relevant.

Response: Agreed. We now point to the practical relevance of our study. *Introduction.* Paragraphs 7 and 8

The standard deviations for participants' average concerts and recorded albums suggest that the variance within these two demographics is relatively high (p. 2, lines 54-56). Were there any differences in sequencing principles between participants who were more of less experienced in album releases or concerts?

Response: As we did not have a working hypothesis there, we did not run any test involving demographic factors. Upon publication, we will make our entire dataset available, and this could be tested by researchers who have theoretical motivation to look for differences in track sequencing based on demographic factors. We have now added a subsection referring to this. *Discussion. Limitations. Demographics and levels of musicianship*. Paragraph 1

I felt that the Methods section missed a few small, but relevant, details. For instance, while I can understand the reasoning behind using jazz and classical music to control for potential instrumentation effects in sequencing practices, I missed why it was necessary to come up with 3 sets of 5 songs in the first place. What was the purpose of comparing the 3 sets?

Response: Yes, our intention was to provide more generalizable results, and to compare possible sequencing differences that would occur given different sets of songs. Ideally, we would work with as many sets as possible, but for time and effort constraints, we decided to go with 3 sets of songs. This is now explained in more detail *Materials and methods. Stimuli.* Paragraph 2.

Next, in the selection of stimuli for set 1, what made a 60 second segment "representative" of a particular track (p. 3, lines 87-88)? Compositionally, e.g., in terms of musical features, were these 60 second segments similar to those in sets 2 and 3?

Response: We did not compare the features of individual segments with those of the entire tracks. Instead, segment representativeness was assessed perceptually by the authors. We acknowledge that this is not a systematic approach and that audio thumbnailing methods could have been used for segment selection.

However, we argue that this is not particularly relevant to the research question posed in the paper, where our primary goal was to evaluate how certain features influence sequencing choices made by musicians. Thus, even if a given segment is not fully representative of the entire track, it still allows us to analyze how features of that particular segment impact sequencing decisions. We now specify this in further detail Materials and methods. Stimuli. Paragraph 5.

Several measures of dissimilarity between consecutive tracks were calculated (p. 5, lines 169-191) based on different (combinations) of audio features. However, their purpose was not immediately clear to me. Could you please elaborate what information these dissimilarity metrics add beyond the initial ANOVA of feature values?

Response: In the Introduction. Paragraph 3, we expose some anecdotal quotes stating, for instance, that consecutive songs should not be similar in terms of tempo and key. Additionally, answers given to items B, F and H address the issue of similarity between consecutive tracks. The dissimilarity scores were thus calculated as a way to check if there are patterns of feature change between consecutive tracks. This is now specified more clearly. Materials and methods. Feature analysis. Paragraph 6.

Furthermore, with regards to the computation of these dissimilarity measures, why was overall audio feature and tempo dissimilarity calculated via Euclidean distance, while key strength profile (dis)similarity was calculated via Pearson correlations? I feel that you must have had a clear reason for doing this, and it would be helpful if this reasoning were further explained.

Response: Good question. Different from the other features, which are expressed by single values of e.g., valence and arousal, the key strength profile is represented by a vector of 24 values (representing the strength of each possible key in major and minor modes). Computing the Euclidean distance between these vectors would give us an idea of key-strength difference, rather than purely key-difference. In other words, Euclidean distance is sensitive to scaling (i.e., different key-profile strengths), while Pearson's r is only sensitive to the shape of key-profiles from each segment. This is now briefly justified in *Materials and methods. Feature analysis.* Paragraph 8.

The manuscript reports a statistically significant interaction effect between position and feature (p. 7, lines 229-230). Were any post-hoc tests conducted to further examine this statistically significant interaction?

Response: We have not conducted post-hoc t-test tests. However, we have now conducted a post-hoc ANOVA to test for the significance of U-shapes. We compared a quadratic versus a linear model that explains feature values across positions and feature types. If, in fact, the U-shape had statistical significance, a quadratic model would be a better fit than a linear one. This is now shown to be the case, and is specified in *Results. Feature analysis* - paragraph 3.

Perhaps this is a bit of a layman's perspective on the matter, but I feel that songs are rarely experienced as individual features, rather a combination multiple. Thus, while the results and subsequent discussion points highlight the role of individual audio features in track sequencing principles, I wonder whether musicians deliberately consider individual features as opposed to a combination of several in their sequencing choices. Is this something that can be inferred from the data obtained for the manuscript, and, if so, can the Discussion be expanded to address this?

Response: Interesting question. In fact, music perception tends to be highly complex, and it usually depends on a wide variety of features. In order to address this issue, we used valence and arousal metrics from MIRtoolbox, which precisely offer a linear combination of 5 different features that contribute to the

perception of emotion in music. Of course, one could include an unlimited amount of interactions between other features, but for the purpose of this paper, we decided to stick to features that have a somewhat facilitated interpretability. Responding to requests from the editor, we also included some more information about how valence and arousal were computed, validated, and used in past literature *Materials and methods. Feature analysis*. Paragraph 3.

This is a slightly nit-picky comment, but if recruitment was done in three separate stages (p. 4, lines 115-116), one per set of songs, is it still accurate to say that participants were randomly assigned to each set of songs (p. 3, line 92)? Here too, were there any pronounced differences in demographics between participants at each stage?

Response: As the recruitment strategy, target group and online platforms did not change between the 3 stages, we thought of reporting it as a random assignment of participants, but we realize that there is space for questioning here. The word "randomly" is now removed. *Materials and methods. Procedures*. Paragraph 1. With regards to demographics, we now report all statistics separately for each set. *Supporting information. Mean and standard deviation of participants' musical experience across sets*. We would still prefer to avoid hypothesis testing in this regard, as we do not have a working hypothesis, or theoretical motivation to do so. Of course, we are ready to discuss this further, if needed.

For consistency, I suggest changing the "Survey" header (p. 7, line 240) to "Questionnaire" (p. 3, line 97; or the other way around).

Response: Done.

Reviewer 2

Participants: Could more details be given about these professional musicians and music producers who were recruited? For example, what type of musical training did they have? I like that the track sequencing focused on little-known instrumental jazz and classical music, however, I would like to know more about these participants. Do they have a lot of experience with jazz or classical music? Do we think this may alter how they sequence the tracks?

Response: Responding to the requests of reviewer 1, we added additional information about music participants' background *Supplementary information. Mean and standard deviation of participants' musical experience across sets*. We did collect additional music training information (i.e., main genre of interest and type of formal training). However, as responses were collected in textual format, where participants could respond freely, analyzing these responses will require considerable text-parsing and interpretation efforts. As we do not have a working hypothesis to suggest that types of musicianship affect sequencing regularities, we did not involve these demographic factors in any of our analysis. This is now specified in further detail *Discussion. Limitations. Demographics and levels of musicianship* Paragraph 1.

I understand using 5 tracks for this experiment as that seems like a reasonable amount for participants to handle sequencing. However, from an ecological validity standpoint, most albums have more than that, say 10-20 tracks. How did the authors settle on 5 tracks per set?

Response: Our previous study (Neto et al., 2024) found that sequencing regularities remained consistent across different album lengths, suggesting that this variable was not a primary factor to influence track sequencing. However, we acknowledge that exploring this variable with respect to sequencing strategies would be an interesting avenue for future research. This consideration is now exposed with more detail in Discussion. Limitations. Number of Tracks.

I really liked using the survey to investigate agreement with statements about perspectives on sequencing. I think future studies might look at WHY participants feel this way. Do we think there is something "universal" or general about auditory sequencing? In other words, is there a certain "story" that is told when sequencing tracks? Also, I imagine results may vary a lot if these were songs with lyrics. I think some of this could be mentioned, even if it's exploratory, in the discussion to ground why it's important to think about album sequencing (i.e. the significance).

> **Response:** Absolutely agreed. I personally believe that there might be something universal about the effect of track sequencing. In more general terms, what you perceive now is likely to influence what you perceive later. If that is the case, musicians might be trying to fulfill some perceptual goal with a given sequencing strategy. We discuss this in the section *Album sequencing. Emotional arcs and storytelling. Why do musicians care about song sequencing?* In our research project, the next step is to directly evaluate the perceptual implication of different track sequences. We hope to bring some light into this issue soon.
>
> I agree with your hypothesis about the influence of lyrics. In this paper we cited studies that looked at the evolution of lyrics, but only within the boundaries of one song. One could definitely look at the distribution of lyric-related features throughout an album or a concert. This would fall a bit out of a strictly auditory realm, and will likely involve linguistic expertise. Nevertheless, we now point to this possible venue of research in Discussion. Album sequencing, emotional arcs and storytelling. Song lyrics. Paragraphs 1, 2 and 3.

Reviewer 3

Clarify how the "inverted U-shape" of tempo and the opposite pattern of valence, arousal, and loudness were measured. It might be helpful to mention the use of MIRtoolbox here to strengthen the methodological transparency.

> **Response:** Yes, other reviewers have also pointed to this issue. We have now tested the U-shapes by comparing a quadratic versus a linear fit. Details in *Results. Feature analysis* - paragraph 3. The usage of MIRtoolbox has been specified with further detail in *Introduction. Paragraph 9*; *Materials and methods. Feature analysis. Paragraphs 3 and 4*.

The introduction mentions a previous study involving 51,010 published albums. It would be useful to briefly explain whether the current study expands on those findings or addresses a different aspect of track sequencing.

> **Response:** Yes, the current study improves on the previous by 1) using the open source MIRtoolbox to extract features, rather than the proprietary Spotify ones; 2) by evaluating the agreement of different musicians about how to sequence the same set of songs; and 3) by conducting an exploratory survey to understand how musicians explicitly think about song sequencing strategies. This is explained in detail in the *Introduction. Paragraph 9.*

The rationale for selecting only jazz and classical genres is well-explained, but the authors might consider addressing whether findings could generalize to more popular or rhythmically consistent genres, such as pop, rock, or EDM.

> **Response:** Here we make reference to our previous study (Neto et al., 2024) to justify the reason why we think musical genre may not be so relevant for track sequencing (Materials and methods. Stimuli.

Paragraph 2; *Introduction. Paragraph 6; Figure 1)*. Furthermore, our present study did not find an effect of set (*Discussion. Paragraph 1)*, which suggests that sequencing principles operated in comparable ways throughout jazz and classical. Even though we have evidence to believe that sequencing principles might generalize across different genres, we still point to this factor as a possible venue for future investigation. Discussion. Limitations. Demographics and levels of musicianship.

The calculation of Positional Regularity (PR) is detailed, but it might be clearer to provide an example illustrating how PR reflects consensus among participants.

Response: We give the following example of PR: If 90% of participants choose track A as the opening of an album, PR is high. If all tracks have been chosen with the same probability for a given position (20%), PR is low. *Materials and methods. Sequencing analysis. Paragraph 3.*

While the ANOVA results are statistically significant, the η^2 values suggest small effect sizes. It would be helpful to discuss whether these effects, while significant, are practically meaningful in real-world album production.

Response: We believe that the meaningfulness of sequencing regularities would be best explored as a perceptual investigation addressing how different sequencing strategies affect the experience of the listener. Even if feature differences between album positions are small, it is possible that these have a huge perceptual effect, or no effect at all. Unfortunately, we can only hypothesize about such effects in the present study, as we have done in *Discussion. Why do musicians care about song sequencing?* Paragraphs 1, 2, 3 and 4. In any case, we now explicitly point to the fact that our η^2 suggest small effect sizes in our feature analysis. *Results. Feature analysis.* Paragraph 1, and we mention this again in *Discussion. Why do musicians care about song sequencing?* Paragraphs 1.

The comparison between emotional arcs in album sequencing and literary works is compelling but somewhat speculative. Consider adding a disclaimer emphasizing that while the patterns are similar, the underlying cognitive mechanisms might differ.

Response: We agree. This is now specified in *Discussion. Album sequencing, emotional arcs and storytelling. Literary works. Paragraph 4.,* as well as in Discussion. Album sequencing, emotional arcs and storytelling. Song lyrics. Paragraphs 2 and 3.

The conclusion effectively summarizes the findings but could benefit from emphasizing the study's implications for music producers and platforms like Spotify or Apple Music, where track sequencing might influence user experience.

Response: This has also been pointed out by another reviewer, and we have now indicated possible practical applications studying track sequencing might bring. Basically, we see track sequencing investigation as a possible way to inform automatic playlist generation, next-track recommendation systems, and DJ-set creation. *Introduction.* Paragraph 8. *Conclusion.* Paragraph 1.
